# Research advances on the role of programmed endothelial cell death in sepsis

**DOI:** 10.1038/s41420-025-02728-x

**Published:** 2025-10-06

**Authors:** Yichen Bao, Xingpeng Yang, Pengyue Zhao, Xiaohui Du

**Affiliations:** 1 Department of General Surgery, First Medical Center of the Chinese PLA General Hospital, Beijing, China; 2 School of Medicine, Nankai University, Tianjin, China

**Keywords:** Infection, Apoptosis, Diagnostic markers

## Abstract

Sepsis is a life-threatening systemic inflammatory response syndrome triggered by infection, characterized by a dysregulated host immune response to pathogenic organisms and associated with substantial morbidity and mortality. According to the most recent sepsis guidelines, effective monitoring and therapeutic strategies remain insufficient, leading to suboptimal patient outcomes. Endothelial cells (ECs) constitute a critical pathophysiological nexus in sepsis pathogenesis, wherein their dysregulation disrupts both microvascular homeostasis and endothelial barrier competence. During sepsis, aberrant activation of programmed cell death (PCD) pathways in ECs induces both structural and functional disruptions, thereby enhancing vascular permeability, causing hemodynamic instability, promoting systemic circulatory dysfunction, and compromising tissue perfusion. These pathophysiological derangements potentiate a vicious cycle of systemic inflammatory amplification, exacerbate disseminated intravascular coagulation, and culminate in lethal multiple organ dysfunction syndrome. This comprehensive review systematically evaluates contemporary insights into the molecular pathophysiology of PCD pathways in endothelial cells during sepsis, with particular emphasis on their mechanistic interplay and therapeutic implications, providing an in-depth understanding of their contributions to sepsis pathophysiology. Additionally, we explore the potential of key PCD-associated molecules as biomarkers for monitoring and evaluating vascular function and permeability in septic patients. Finally, we discuss the current state of drug development targeting ECs’ PCD and their prospective therapeutic implications for sepsis, offering valuable insights for future basic research and clinical applications.

## Facts

- Programmed endothelial cell death represents a pivotal pathophysiological mechanism contributing to endothelial structural disruption, vascular homeostasis dysregulation, and the exacerbation of inflammatory responses, thrombus formation, and vascular remodeling.
- Programmed endothelial cell death contributes to glycocalyx degradation, coagulopathy, and inflammatory dissemination, ultimately compromising tissue perfusion and organ function.
- Programmed endothelial cell death acts as a novel biomarker for early hemodynamic monitoring in sepsis and provides novel therapeutic intervention targets for sepsis.

## Open questions

- What programmed endothelial cell death modalities contribute to the pathophysiology of sepsis?
- What are the mechanisms by which programmed endothelial cell death contributes to the pathophysiological progression of sepsis?
- What are the potential applications of modulating endothelial programmed cell death in the diagnosis and treatment of sepsis?

## Introduction

Sepsis represents a life-threatening systemic condition characterized by infection-triggered organ dysfunction resulting from dysregulated host responses, which imposes a significant worldwide disease burden with remarkably consistent epidemiological patterns across geographic regions. A seminal 2020 Lancet epidemiological analysis demonstrated sepsis-related mortality accounted for 19.7% of worldwide mortality between 1990 and 2017, equating to approximately 11 million annual deaths [1]. Clinically, 30–50% of cases progress to the stage of multiple organ dysfunction syndrome (MODS), with mortality rates exceeding 40% in refractory septic shock cohorts [2, 3]. Microbial pathogens, predominantly Gram-negative bacteria and respiratory viruses, invading through predominant infection foci (pulmonary, intra-abdominal, genitourinary, and bloodstream) initiate this catastrophic cascade [4]. While advances in antimicrobial stewardship and extracorporeal organ support have improved survival rates, sepsis persists as a formidable biomedical challenge. Critical knowledge gaps persist in two domains: the first one is the absence of temporally sensitive diagnostic signatures for early detection, particularly pre-symptomatic biomarkers; and the second one is the paucity of mechanism-based therapies targeting immunometabolism reprogramming rather than generic inflammation suppression [5].

Malperfusion syndromes and hemodynamic instability constitute core pathomechanistic drivers of sepsis-induced multiorgan failure. ECs as critical pathophysiological determinants of vascular homeostasis, orchestrate immunothrombotic balance and microcirculatory competence—processes catastrophically disrupted during septic progression [6]. The septic milieu triggers EC injury through coordinated activation of programmed death modalities (apoptosis, pyroptosis, ferroptosis), initiating a lethal triad of cytokine storm amplification, immunothrombotic dysregulation, and barrier disintegration. These pathomechanisms culminate in capillary leakage, distributive shock, and tissue hypoxia, cardinal features of irreversible organ injury [7]. Recognizing this endothelial-centric paradigm, the 2023 Surviving Sepsis Campaign guidelines have formally incorporated endothelial protection metrics into therapeutic algorithms [8]. Nevertheless, several knowledge gaps persist: firstly, the spatiotemporal regulation of multifarious PCD and its crosstalk with ferroptotic/autophagic pathways remain unmapped in septic endotheliopathy; besides, preclinically validated PCD inhibitors show discordant efficacy in clinical trials, necessitating cell type-specific delivery systems [9]. Finally, Current biomarkers fail to differentiate adaptive EC stress responses from terminal death commitment [10]. This critical appraisal synthesizes emerging concepts of endothelial death plasticity while proposing a targeted research agenda to harness EC resilience pathways for sepsis management.

This review systematically summarizes recent advances in the molecular mechanisms and regulatory networks of PCD (apoptosis, necroptosis, pyroptosis, and ferroptosis, etc.) in endothelial cells during sepsis. By elucidating the pathophysiological roles of ECs’ PCD in the progression of sepsis, we propose to investigate the potential application of PCD-related biomarkers and small-molecule compounds for monitoring of hemodynamic stability in critically sepsis patients, assessing therapeutic efficacy, and predicting outcomes in septic patients. Additionally, we critically evaluate current developments in pharmacological agents targeting PCD and their potential for clinical translation. Through the integration of these multifaceted perspectives, we aim to identify novel therapeutic targets for sepsis management, thereby establishing a comprehensive framework for basic research and clinical applications in this domain.

## Programmed cell death in endothelial cells

**Fig. 1:**
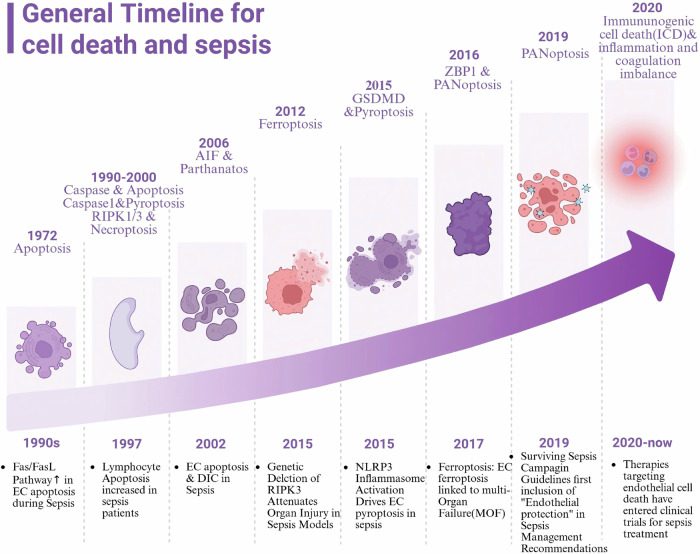
The evolution of the PCD concept. Following biologists’ observations of cell death phenomena, distinct forms of programmed cell death (PCD) have been successively identified, including apoptosis (1972), pyroptosis (1992), necroptosis (2000), and ferroptosis (2012), among others. The key regulatory molecules and mechanisms underlying these processes were gradually elucidated, revealing their interconnected nature rather than independence. This culminated in the proposal of PANoptosis in 2019, emphasizing the integration of multiple PCD pathways. Concurrently, endothelial cells' (ECs) PCD has been recognized as pivotal in sepsis pathogenesis. Recent guidelines (2019) for the first time incorporated “endothelial protection” into sepsis management, and targeting ECs’ PCD mechanisms has emerged as a key focus in current therapeutic research.

The conceptualization and modalities of PCD have continuously advanced in tandem with technological innovations and an enhanced understanding of human diseases (Fig. 1). As early as the mid-19th century, embryologists first identified the morphological characteristics of PCD, recognizing it as a fundamental mechanism for host defense against infections and cellular turnover. In 1964, Richard Lockshin and colleagues first introduced the concept of “programmed cell death”, which was subsequently followed by Kerr et al.’s seminal 1972 publication on the term “apoptosis” in the context of cancer [11]. Over time, the molecular elucidation of these processes has expanded the scope of PCD research, bringing additional modalities, such as pyroptosis and necroptosis, into focus due to their pivotal roles in various human diseases. These PCD pathways are now recognized as essential mechanisms in the pathogenesis of sepsis. Moreover, emerging concepts such as dependency-driven cell death and metabolism-associated cell death have begun to fill critical gaps in the understanding of PCD mechanisms [12]. Notably, the introduction of the term “PANoptosis” in 2019 marked a conceptual breakthrough in understanding the crosstalk and molecular interdependencies between distinct PCD pathways. At present, the precise mechanisms of PANoptosis represent a cutting-edge area of research [13].

ECs’ PCD represents a pivotal pathophysiological mechanism contributing to endothelial structural disruption, vascular homeostasis dysregulation, and the exacerbation of inflammatory responses, thrombus formation, and vascular remodeling [14]. The initiation of ECs’ PCD is governed by a complex array of both endogenous and exogenous factors, including pathogen infections with subsequent endotoxin release, alterations in hemodynamic shear stress, disturbances in glucose and energy metabolism, and exposure to pharmacological agents or toxins [15]. These pathogenic stimuli activate various pathogen-associated molecular patterns (PAMPs) and damage-associated molecular patterns (DAMPs), triggering distinct PCD pathways in endothelial cells [16]. The resulting endoplasmic reticulum (ER) stress and mitochondrial dysfunction release DAMPs, particularly fragmented deoxyribonucleic acid (DNA) and reactive oxygen species (ROS). The ensuing ROS burst serves to upregulate receptor signaling and gene expression linked to PCD pathways. Importantly, mitochondrial dysfunction and oxidative stress represent pivotal regulatory nodes governing ECs’ PCD progression. During sepsis pathogenesis, multiple PCD pathways can coexist and engage in cross-regulatory interactions [17].

Mitochondria generate energy for cellular processes. Their inner and outer membranes maintain barrier function, while respiratory chain complexes I–V on the inner membrane sustain the electrochemical gradient and drive adenosine triphosphate (ATP) production, while also participating in redox homeostasis, Ca²⁺ buffering, and the initiation of apoptosis, among other functions. Mitochondrial quantity and quality are regulated through autophagy, fusion, and fission, ensuring their renewal [18]. Under physiological conditions, ECs maintain redox homeostasis via a balanced antioxidant/pro-oxidant system, encompassing ROS coupled with superoxide dismutase (SOD), the glutathione/glutathione reductase system (GSH/GR), the thioredoxin/thioredoxin reductase system (Trx/TrxR), and non-enzymatic antioxidants (vitamins A, C, and E) [19]. During pathogen infection, uncoupled endothelial nitric oxide synthase (eNOS) and upregulated inducible nitric oxide synthase (iNOS) activity led to excessive reactive nitrogen species (RNS). Additionally, upregulated nicotinamide adenine dinucleotide phosphate hydrogen (NADPH) oxidase expression, iron overload, and mitochondrial electron transport chain (ETC) dysfunction contribute to the generation of ROS, including superoxide radicals. Concurrently, the decline in antioxidant systems (e.g., sirtuin proteins) disrupts redox balance, creating a highly oxidative stress environment. RNS and ROS can further combine to form toxic derivatives that inhibit mitochondrial complexes and key enzymes, induce lipid peroxidation, and damage DNA, proteins, and other biomolecules [18, 20]. Responding to inflammatory, caspase-1/11, and toll-like receptor 4 (TLR4) in immune cells suppresses the adenosine 5’-monophosphate (AMP)-activated protein kinase (AMPK)-Unc-51-like autophagy activated kinase-1 (ULK1) autophagy regulatory pathway, impairing mitochondrial clearance. Excessive mitochondrial fission causes accumulation of damaged mitochondria, upregulates Bcl-2-associated X protein/Bcl-2 homologous antagonist killer (Bax/Bak), and induces mitochondrial membrane pore formation and outer membrane permeabilization (MOMP). This releases mitochondrial DNA (mtDNA) and oxidized mtDNA (ox-mtDNA) into the cytosol, which act as DAMPs, activating NLR family pyrin domain containing-3 (NLRP3) inflammasome assembly, toll-like receptor 9 (TLR9), and the cyclic GMP-AMP synthase (cGAS)-stimulator of interferon genes (STING) pathway, triggering apoptosis, pyroptosis, and inflammation [18]. Furthermore, mtDNA activates Z-DNA binding protein 1 (ZBP1), mediating PANoptosis in endothelial cells [21]. Oxidative stress, metabolic reprogramming, and inflammatory byproducts function as DAMPs, triggering programmed cell death (apoptosis, pyroptosis, necroptosis, and ferroptosis) in endothelial cells [22, 23]. Multiple factors, such as organelles (mitochondria, lysosomes) and microenvironmental conditions (e.g., ROS), regulate diverse forms of cell death, highlighting its complexity through interwoven signaling pathways and their crosstalk [24].

### Apoptosis and parthanatos

Apoptosis is distinguished by activating caspase and the formation of apoptotic bodies, initiating a proteolytic cascade resulting in DNA fragmentation and the degradation of essential regulatory proteins while preserving plasma membrane integrity, thus representing an active and regulated cell death process as well as a component of the normal immune response [25]. Apoptosis is triggered by intracellular (e.g., genotoxic stress) or extracellular stimuli (e.g., death-receptor ligands), comprising extrinsic and intrinsic pathways. The extrinsic (death-receptor-dependent) pathway initiates when tumor necrosis factor receptor (TNFR) superfamily members bind their cognate TNF ligands [26]. Fas ligand (FasL) binds to Fas-associated death domain protein (FADD) and pro-caspase-8, forming the death-inducing signaling complex (DISC). Caspase-8 autoactivation in DISC activates effector caspases-3/6/7, which degrade cellular substrates (e.g., proteins, DNA) to execute apoptosis [27, 28].

The intrinsic pathway (mitochondrial pathway) is activated during sepsis by hypoxia, Ca²⁺ overload, and oxidative stress, etc. Under these conditions, pro-apoptotic B-cell lymphoma-2(Bcl-2) family members Bax/Bak embedded in the mitochondrial outer membrane become activated, neutralizing anti-apoptotic proteins such as Bcl-2 and forming membrane pores. This leads to MOMP, resulting in the release of apoptogenic factors, including cytochrome c (Cyt-C), Second mitochondria-derived activator of caspases (SMAC), and the mitochondrial serine protease Omi/HtrA2 into the cytosol [15, 29]. Cyt-C binds apoptotic protease-activating factor 1 (Apaf-1), inducing oligomerization, while SMAC inhibits inhibitor of apoptosis proteins (IAPs), relieving caspase suppression. The Apaf-1 complex recruits pro-caspase-9 to form the apoptosome, enabling caspase-9 autoactivation, which then activates effector caspases-3/6/7 to execute apoptosis [28, 30]. Omi/HtrA2 exacerbates mitochondrial damage and oxidative stress by modulating mitochondrial stress proteins such as C/EBP homologous protein (CHOP) and fusion proteins, including Optic atrophy 1. In type II cells (e.g., hepatocytes), the extrinsic pathway requires caspase-8-mediated amplification for apoptosis induction. Caspase-8 cleaves BH3-interacting domain death agonist (Bid) to generate truncated Bid (tBid), which activates pro-apoptotic Bcl-2 proteins, inducing MOMP and intrinsic pathway activation [28, 31].

Apoptosis is essential for innate immunity during infection, functioning as an essential mechanism for the host to eliminate infected cells and control infection progression. Unlike other forms of cell death, apoptotic execution does not result in the release of cellular debris that could amplify inflammatory responses. However, complex crosstalk exists between apoptosis and inflammatory pathways [32]. Inflammatory mediators (e.g., lipopolysaccharide (LPS)-interleukin-6 (IL-6)), signaling pathways (e.g., programmed death-ligand 1 (PD-L1)-hypoxia-inducible factor 1α (HIF-1α), nucleotide-binding oligomerization domain (NOD)-like receptor X1 (NLRX1)-tumor necrosis factor-α (TNF-α)), and IκB degradation promote nuclear factor-κB (NF-κB) nuclear translocation and Yes-associated protein (YAP) phosphorylation, ultimately upregulating pro-apoptotic genes [33]. MOMP degrades IAPs under caspase inhibition, activating NF-κB and caspases-1/8 to process Interleukin-1 beta/18(IL-1β/IL-18). MOMP additionally releases the fragmented mtDNA, subsequently catalyzing the conversion of GTP/ATP to cyclic GMP-AMP (cGAMP). This metabolite activates the STING pathway, ultimately activating NF-κB and promoting type I interferon gene transcription. Apoptosis is an ATP-dependent process. During sepsis, LPS upregulates 6-phosphofructo-2-kinase/fructose-2, 6-bisphosphatase 3 (PFKFB3) to enhance EC glycolysis, providing oxygen-independent ATP for apoptosis while concurrently promoting EC migration and NF-κB activation, thus exacerbating inflammation [34].

Parthanatos is distinct from apoptosis, necroptosis, and autophagy, etc. It is mediated by DNA damage-induced hyperactivation of poly (ADP-ribose) polymerase 1 (PARP1), termed PARP1-dependent cell death. Both apoptosis and parthanatos are triggered by severe DNA damage, oxidative stress, and mitochondrial dysfunction, with the mode of death determined by stress intensity and duration. However, apoptosis involves Cyt-c release and caspase activation, whereas parthanatos features apoptosis-inducing factor (AIF) release, causing extensive DNA fragmentation [35]. Initially identified as a novel regulated form of neuronal cell death by Ted and Valina Dawson, parthanatos has since been implicated in brain ischemia, N-methyl-D-aspartate (NMDA) excitotoxicity, and neurodegenerative disorders such as Parkinson’s disease. In neurons, NMDA receptor activation and glutamate-induced Ca²⁺ influx stimulate neuronal nitric oxide synthase (nNOS), producing nitric oxide (NO). NO reacts with superoxide (O₂⁻) to form peroxynitrite (ONOO⁻), inducing DNA damage and macromolecular injury. This triggers PARP1 activation, initiating the PARP1-NAD + /ATP depletion-cell death cascade [36].

In sepsis, DNA damage and mtDNA release induce PARP1 overactivation, triggering AIF release and nuclear translocation. Nuclear AIF binds macrophage migration inhibitory factor (MIF), forming an AIF/MIF complex that mediates chromatin condensation, DNA fragmentation, and caspase activation, while depleting NAD + /ATP to execute cell death [37, 38]. Key features of Parthanatos include: (1) Caspase independence; (2) Mitochondrial membrane depolarization and secondary ROS generation; (3) Dependence on calcium signaling; (4) Resistance to Bcl-2-mediated cytoprotection [36]. Inhibition of parthanatos may shift cell death toward the apoptotic pathway [36]. Caspase inhibition/impairment triggers parthanatos as an alternative cell death pathway. This complementary relationship positions Parthanatos as a potential therapeutic target in apoptosis-resistant tumors [35]. Excessive PAR accumulation also promotes ferroptosis, while TNF-α can regulate necroptosis via ATP depletion and PARP1 activation. However, its precise role in endothelial cells during sepsis remains unclear. PARP1 deficiency suppresses angiotensin II and upregulates eNOS expression, improving endothelial function [7]. Furthermore, Xue et al. demonstrated that erlotinib inhibits LPS-induced parthanatos by suppressing TLR4 expression on macrophages [39]. Similarly, Yan et al. developed Olaparib (OLA)-engineered nanocapsules, which exhibit broad-spectrum anti-inflammatory effects and prevent sepsis-induced intestinal injury by inhibiting parthanatos [40]. These findings highlight the therapeutic potential of targeting parthanatos in sepsis management.

### Inflammatory cell death (pyroptosis and necroptosis)

During sepsis progression, both cytokine storm and systemic inflammatory response syndrome (SIRS) are characteristic complications that significantly contribute to multiorgan dysfunction and mortality. The initiation phase, characterized by inflammation formation and dissemination, comprises pyroptosis and necroptosis, representing a critical pathological mechanism. These processes share plasma membrane disruption, resulting in intracellular component release into the interstitial space, which amplifies local inflammation, disrupts immune homeostasis, and worsens tissue damage [41].

Pyroptosis hallmarks include inflammasome assembly/activation, plasma membrane pore formation, and proinflammatory cytokine maturation [42]. LPS binding to TLR4 upregulates myeloid differentiation primary response 88 (MyD88)/TIR-domain-containing adapter-inducing interferon-β (TRIF) expression [43], while ROS generation promotes thioredoxin-interacting protein (TXNIP) dissociation, upregulating Cyt-c release and subsequent NLRP3 inflammasome assembly. NLRP3 activates the adapter apoptosis-associated speck-like protein (ASC) and caspase-1, forming the inflammasome complex. This complex cleaves gasdermin D (GSDMD), releasing its N-terminal fragment (N-GSDMD), which integrates into the plasma membrane to form lytic pores, causing osmotic swelling and cytolysis. Concurrently, NF-κB activation promotes the maturation of IL-18 and IL-1β, which are secreted through these membrane pores, upregulating EC adhesion molecules and disrupting VE-cadherin localization, thereby increasing vascular permeability. LPS can also directly upregulate caspase-11 (non-canonical pathway), where caspase-11-mediated GSDMD cleavage contributes to inflammatory blood-brain barrier disruption [23]. Hyperglycemia, dyslipidemia, and nicotine can elevate ROS levels in ECs [43], upregulating high mobility group box 1(HMGB1) and potentiating pyroptosis owing to the inflammasome. Functional blockade of human neutrophil peptides 1–3(HNP1-3) has been shown to mitigate pyroptosis [44]. Following pyroptosis, the release of cellular contents and inflammatory cytokines amplifies DAMPs, altering cell adhesion and propagating inflammation, ultimately leading to vascular hyperpermeability and cytokine storm formation. Additionally, this process can activate the coagulation cascade, resulting in coagulopathy [45].

Necroptosis is a PCD mechanism resembling necrosis, characterized by its energy-independent nature. Mixed lineage kinase domain-like (MLKL) phosphorylation by the receptor-interacting protein kinase-1/3 (RIPK1/3) complex is essential for the necroptosis pathway. In sepsis, tumor necrosis factor receptor 1(TNFR1) and TLR3/4 activation recruit RIPK1/RIPK3, culminating in MLKL phosphorylation. MLKL oligomerizes into a β-barrel-shaped necrosome complex that stably integrates into the lipid bilayer, causing ion dysregulation, massive water influx, and terminal plasma membrane disruption [37, 46]. Consequently, intracellular components, chemokines, inflammatory cytokines, and potassium ions are released, exacerbating inflammation, coagulation dysfunction, and even MODS [37]. Experimental studies demonstrate that genetic deletion of MLKL/RIPK, under pretreatment with either anticoagulant or non-anticoagulant heparin, protects the mouse model from TNF-α-induced coagulopathy or SIRS [47]. Furthermore, emerging evidence indicates that RIPK serves as a critical upstream regulator triggering other forms of PCD [48]. Thus, RIPK inhibition represents a potential target for mitigating SIRS and ameliorating sepsis progression in affected patients [46].

### Metabolic cell death

Metabolic cell death refers to cell death caused by metabolic dysregulation. It is typically associated with dysregulation of specific metabolic pathways and aberrant accumulation or depletion of metabolites, such as excessive consumption or overload of certain nutrients (e.g., glucose, amino acids) or metal ions (e.g., iron and copper), leading to metabolic imbalance-induced cell death. Common forms include ferroptosis, cuproptosis, disulfidptosis, and osteonecrosis, among other pathways [49, 50]. Ferroptosis has been relatively well-elucidated in the pathophysiological mechanisms of sepsis, whereas the roles of other forms of metabolic cell death await elucidation, potentially offering novel therapeutic targets.

Ferroptosis is characterized by iron-dependent lipid peroxidation, iron overload, and glutathione peroxidase 4 (GPX4) downregulation. During sepsis, LPS upregulates the iron channel protein transferrin receptor 1(TfR1), leading to increased Fe²⁺ influx and subsequent overload, resulting in mitochondrial shrinkage, ROS accumulation, excessive depletion of glutathione (GSH) and GPX4, intracellular redox imbalance, and necrotic cell death due to peroxidation of membrane lipids [51]. GPX4 is a key inhibitor of ferroptosis. Ferroptosis promotes infection by supplying nutrients for bacterial proliferation while inducing autoimmunity and immune dysfunction, contributing to sepsis-associated kidney injury, septic encephalopathy, and acute respiratory distress syndrome (ARDS) [52, 53].

Ferrostatin-1 (a lipophilic ROS scavenger) and deferoxamine (DFO) (an iron chelator) exert anti-ferroptotic effects by inhibiting lipid peroxidation and iron accumulation via the p53-xCT-GSH axis [37]. Beyond ferritin degradation pathways, selective autophagy activation can also promote ferroptosis [54]. ZnO nanoparticle treatment activates the autophagy pathway, inducing ferritin degradation in a nuclear receptor coactivator 4 (NCOA4)-dependent manner, leading to Fe^2+^ overload and ferroptosis [55]. Wu et al. demonstrated that lactate accumulation during sepsis stimulates alveolar epithelial cells to undergo mitochondria-associated ferroptosis via the G protein-coupled receptor 81 (GPR81) /histone H3 lysine18 lactylation (H3K18la) /methyltransferase-like 3(METTL3) / acyl-CoA synthetase long-chain family member 4(ACSL4) axis, contributing to acute lung injury (ALI) [56]. While the classical ferroptosis pathway and its role in sepsis are well-established, the interplay between ferroptosis and other PCD mechanisms, as well as potential therapeutic targets, requires further investigation.

### Interplay between ECs’ PCD and PANoptosis

Accumulating evidence indicates that distinct PCD pathways do not function independently but exhibit complex crosstalk and interconnection, although their precise molecular interactions remain incompletely understood [57]. Caspase-8 controls the switch between distinct types of PCD. Inhibition of caspase-8 and FADD can redirect cell fate from apoptosis to necroptosis [58], while caspase-8 also participates in regulating NLRP3 inflammasome formation during pyroptosis. Furthermore, executioner caspases-3 and -7 not only cleave GSDMD to generate its N-GSDMD but also process GSDME, thereby mediating the phenotypic transition from apoptosis to pyroptosis. Conversely, MLKL pore formation during necroptosis may secondarily trigger apoptotic pathways through membrane damage. Additionally, autophagy-dependent ferritin degradation has been shown to induce endothelial cell ferroptosis [37].

**Fig. 2:**
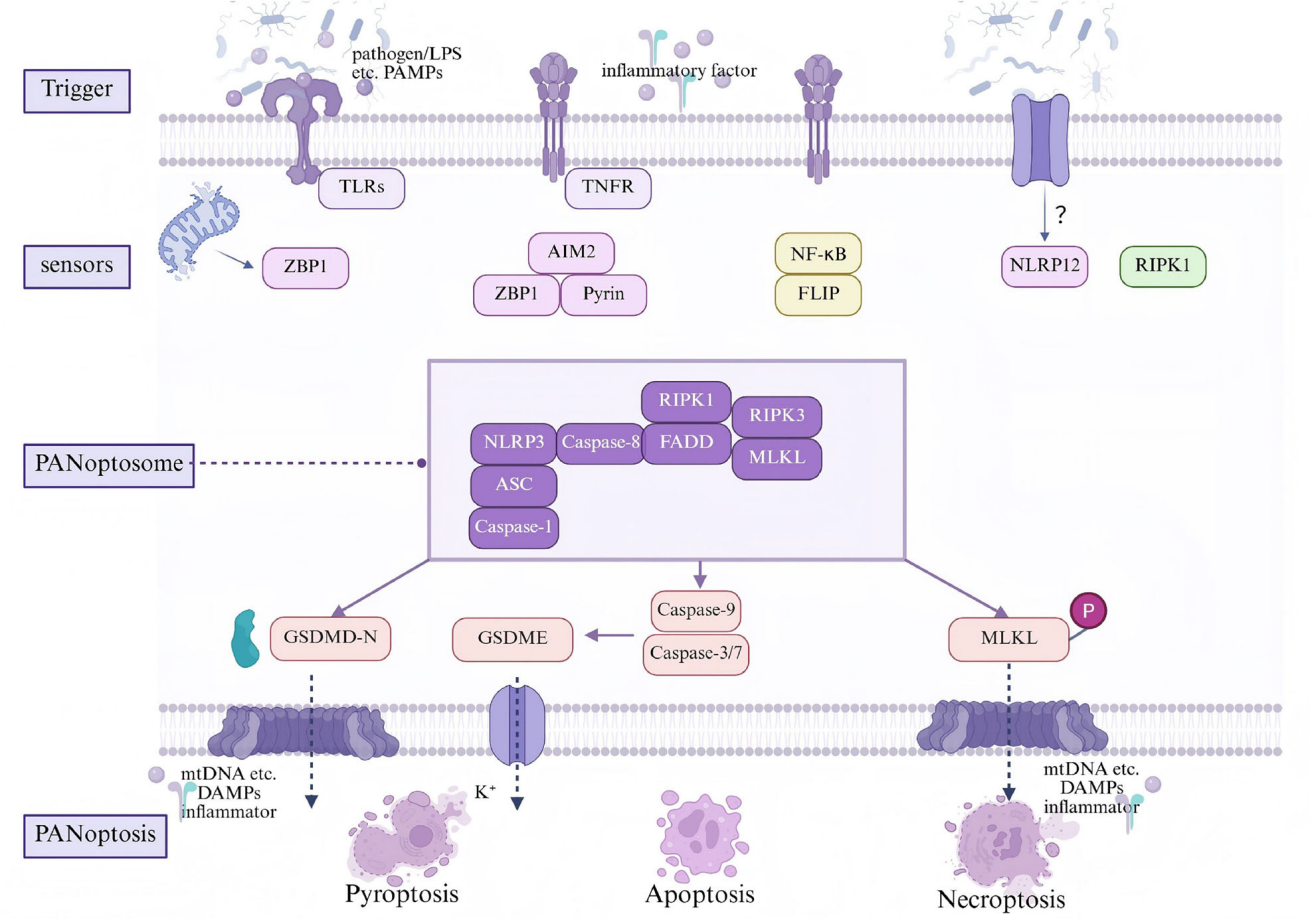
Molecular mechanisms of PANoptosis in ECs during sepsis. PANoptosis is a novel cell death paradigm proposed based on the interference and crosstalk between different programmed cell death pathways. This process is initiated when pattern recognition receptors (PRRs) such as Toll-like receptors (TLRs) and tumor necrosis factor receptors (TNFRs) sense pathogen-associated molecular patterns (PAMPs) or damage-associated molecular patterns (DAMPs). Subsequent activation of downstream sensors, including ZBP1, AIM2, pyrin, NF-κB, RIPK1, and NLRP12, triggers the assembly of PANoptosome complexes. The execution phase involves gasdermin-D N-terminal fragments (GSDMD-N), gasdermin-E (GSDME), and phosphorylated mixed lineage kinase domain-like protein (p-MLKL), integrating into plasma membrane lipids to form transmembrane pores. This leads to the release of cellular contents and DAMPs, ultimately resulting in a composite cell death outcome incorporating features of apoptosis, pyroptosis, and necroptosis.

PANoptosis is a caspase- and RIPK-dependent inflammatory lytic cell death pathway, uniquely regulated by the PANoptosome complex [59]. The conceptualization of PANoptosis has shifted research focus toward the integrated regulation of PCD pathways (Fig. 2). The PANoptosome functions as a master molecular switch for downstream effector activation, with well-characterized upstream regulators including ZBP1, absent in melanoma 2 (AIM2), RIPK1, and NLRP12 [60–62]. During sepsis, pathogen infection and inducers such as TLR4, TNF, FasL, NF-κB, and inflammatory cytokines can bind to cell surface death-associated receptors, activating upstream sensors (e.g., ZBP1) to form the PANoptosome. This complex facilitates the recruitment and activation of RIPK1, RIPK3, MLKL, FADD, caspase-8, NLRP3, ASC, and caspase-1, subsequently triggering phosphorylation of MLKL, maturation of caspase-9/3/7, cleavage of GSDMD, and GSDME processing by caspase-3. These events culminate in a lytic cell death exhibiting hybrid features of apoptosis, necroptosis, and pyroptosis, collectively termed PANoptosis [63, 64]. However, the precise regulatory mechanisms connecting cell surface death receptors to ZBP1 and other sensors, as well as sensor-PANoptosome interactions, require further elucidation.

The involvement of PANoptosis in sepsis pathogenesis has been experimentally validated [65]. Studies demonstrate that neutrophil-derived S100A8/A9 downregulates nuclear respiratory factor 1 (Nrf1) and suppresses expression of NADH dehydrogenase in mitochondrial complex I. Functioning as a transcriptional regulator in concert with coactivators such as PGC-1α, Nrf1 governs the expression of nuclear-encoded mitochondrial genes (NEMGs) involved in: mitochondrial fusion (MFN1/2, OPA1), fission (DRP1, FIS1), mitophagy (FUNDC1), etc. The S100A8/A9-mediated Nrf1 suppression leads to pathological mitochondrial hyperfission and impaired mitophagy, resulting in mitochondrial DNA fragment release that ultimately triggers PANoptosis activation [21, 66, 67]. Gong et al. identified the cold-inducible RNA-binding protein (CIRP)-ZBP1-PANoptosis axis as a critical mediator of lactate-induced pulmonary endothelial cell death in sepsis. Lactate accumulation promotes CIRP lactylation and secretion from macrophages; secreted CIRP then binds TLR4 while competitively activating ZBP1, thereby potentiating ZBP1-RIPK3-dependent cell death [68]. He et al. demonstrated that ursodeoxycholic acid (UDCA) ameliorates sepsis-induced lung injury by suppressing PANoptosis via STING pathway modulation [69]. Table 1 provides a summary of the PCD patterns exhibited by ECs.

**Table 1:** Characterization of the mode of programmed endothelial cell death.

PCD	Main processes and pathways		Cell membrane and nucleus changes	Inflammation	Reference
Apoptosis	Regulatory receptor	Exogenous: death-receptor ligand (Fas-associated death structural domain)/dependent receptor ligand	Nucleus: chromatin condensed and broken, nucleus consolidated, autophagosomes formed; Cytosol: cellular integrity, crumpling, cytoplasmic efflux, and vacuolization of the cytosol membrane.	LPS-IL-6, PD-L1-HIF-α, NLRX1-TNF-α; Promotes IκB degradation, NF-κB nuclear translocation, YAP phosphorylation, and activates apoptotic gene expression.	[15, 18, 25, 28, 33, 34]
		Endogenous: Cyc release from mitochondrial damage, oxidative stress, etc.			
	Effector genes and proteins	Common apoptosis-related genes: Bax, Bcl-2, Bcl-xL, p53, etc.			
		Executioner caspases: caspase-3/6/7			
		Initiator Caspases: caspase-8/9/10			
		Mitochondria-cleave: Cyt-c, SMAC, mtDNA, etc.			
	Main process	Apoptotic vesicle formation; Initiation of protein substrate hydrolysis; DNA and key regulatory protein fragmentation.			
	Effect	Altered vascular permeability; Inflammatory factor release; Impairment of organ function.			
Necroptosis	Regulatory receptor	RIPK1/3	Nucleus: chromatin breaks, DNA fragmentation Cell membrane: rupture of the cell membrane, discharge of cytoplasmic contents leading to inflammation and other reactions	Necrotic apoptosis occurs in response to TNF, Fas, or TRAIL, among others, and has a highly proinflammatory effect	[37, 46–48]
	Effector proteins	MIKL			
	Main process	RIPK1/3 activation, phosphorylation of MIKL, necrotic apoptotic cell membrane rupture, and content release			
	Effect	Altered vascular permeability; the Inflammatory and cytokine storms; Coagulation abnormalities; Organ function impairment			
Pyroptosis	Regulatory receptor	Classical pyroptosis pathway: NLRP3 captures ASC cleavage of GSDMD-N; activation of caspase-1 by LPS-HMGB1, NLRP-1/3, NLRC4	Nucleus: chromatin breaks, DNA fragmentation; Cell membrane: formation of membrane pores, swelling and rupture of the cell, discharge of cytoplasmic contents, triggering inflammatory reactions.	Pyroptosis is a natural immune response triggered by the activation of inflammatory vesicle assembly, which promotes the mature secretion of inflammatory factors such as IL-1β and exacerbates the local inflammatory response	[23, 42–45]
		Non-classical pyroptosis pathway: caspase-11 (murine origin), caspase-4/5 (human origin)			
	Effector proteins	Classical pathway: GSDMD-N integration to form membrane pores; casepase-1/3/9			
		Non-classical pathway: caspse-11, caspase-4/5 (human source)			
	Main process	Inflammatory vesicle NLRP3-ASC-GSDMD-N assembly activation, membrane pore formation, proinflammatory cytokine maturation, and release			
	Effect	Altered vascular permeability. Inflammatory response; Cytokine storm; Coagulation abnormalities; Organ function impairment			
Ferroptosis	Regulatory receptor	GPX4 over depletion, NCOA4 autophagy-dependent iron death	Nucleus: no change Cell membrane: ruptured Mitochondria: outer mitochondrial membrane ruptured and crumpled; mitochondria darkly colored	Activation of JAK-STAT, NF-κB, inflammatory vesicles, cGAS-STING, and MAPK signaling pathways can lead to iron death	[37, 51–53]
	Effector genes and proteins	Ferritin degradation; TFRC/HSPβ1-TFR-1; Nfr2-ROS; NCOA4 activation of the ATG5-ATG7-NCOA4 autophagy pathway, culminating in ferric iron overload			
	Main process	Iron ion-dependent lipid peroxide accumulation, iron overload, and (GPX4) downregulation			
	Effect	Altered vascular permeability; Inflammatory response; Impairment of organ function			
PANoptosis	Molecular mechanisms	PANoptotic vesicles are downstream molecular initiation switches. Currently, the more explicit PANoptotic upstream molecules include ZBP1, AIM2, RIPK1, etc. The known pathways are: S100a8/a9-Nrf1-PANoptosis; (CIRP)-ZBP1-PANoptosis; EIF2AK2-AIM2-PANoptosis, and the specific pathways need to be clarified.	-	NLRP12 drives inflammatory vesicle and PANoptosome activation through IRF1 and TLR2/4 in response to PAMP and TNF, causing cell death and promoting inflammation.	[21, 60–64] [68, 69]

## Mechanisms of ECs PCD in sepsis

**Fig. 3:**
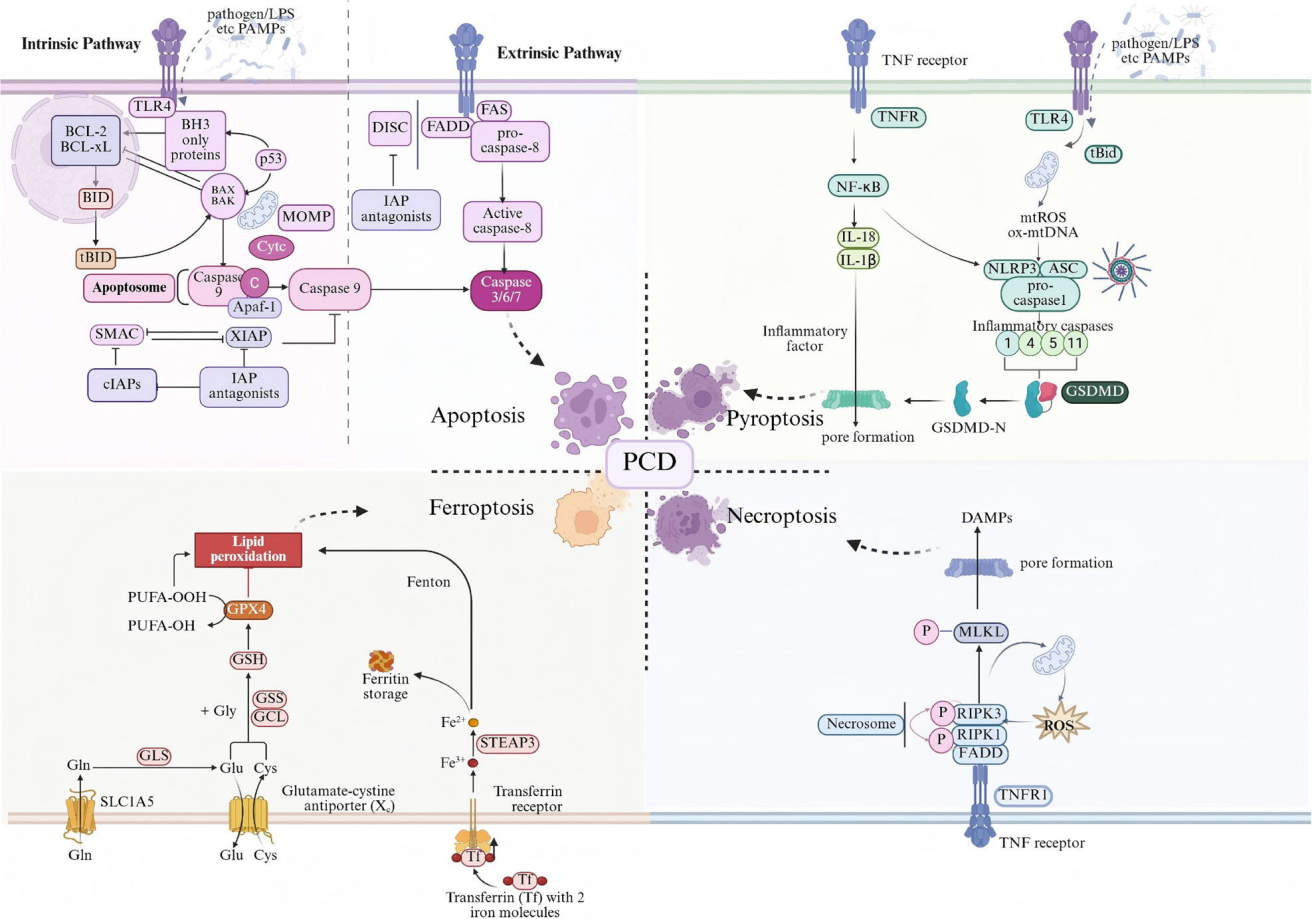
Molecular mechanisms of PCD in ECs during sepsis. In sepsis, endothelial pattern recognition receptors—TLRs sensing PAMPs (LPS, etc.) and TNFRs sensing circulating cytokines (TNF-α, IL-1β, IL-18)—trigger distinct PCD pathways. Apoptosis is initiated by either the mitochondrial route (BAX/BAK-mediated MOMP, antagonized by BCL-2/BCL-xL) or the death-receptor route (ligand-activated TNFR/FAS recruiting FADD, DISC, and caspase-8), culminating in the activation of irreversible caspase cascades. Pyroptosis follows inflammasome assembly: NLRP3 senses ox-mtDNA, mtROS, and NF-κB signaling, assembling with ASC and pro-caspase-1 to generate inflammatory caspases that cleave GSDMD-N, forming membrane pores and releasing inflammation factors. When caspase-8 is inhibited, necroptosis ensues via RIPK1/RIPK3 necrosome assembly and MLKL phosphorylation, producing membrane pores. Ferroptosis is driven by iron-dependent lipid peroxidation: transferrin delivers Fe2⁺, system Xc⁻ imports cystine for GSH synthesis, and GPX4-mediated detoxification of PUFA-OOH. Loss of GPX4 or GSH accumulation of PUFA-OH propagates Fenton chemistry, yielding lethal membrane damage. IAPs, SMAC, and cIAPs modulate caspase activity and final cell fate.

As the intimal lining of both blood and lymphatic vessels, ECs play a fundamental role in vascular endothelial barrier integrity. They are crucial for maintaining vascular permeability, mediating substance transport, regulating immune responses, and modulating hemostasis. During sepsis, PAMP/DAMP-induced ECs PCD causes hemodynamic instability and vascular leakage. Subsequent release of proinflammatory mediators(chemokines and cytokines) and cellular debris, activating coagulation, inflammation, and leukocyte recruitment (Fig. 3). These processes contribute to glycocalyx degradation, coagulopathy, and inflammatory dissemination, ultimately compromising tissue perfusion and organ function. Furthermore, these events can precipitate severe complications, including cytokine storm and disseminated intravascular coagulation (DIC), SIRS, and MODS, which pose a significant mortality risk [70].

### Glycocalyx damage and vascular permeability

The endothelial glycocalyx, a luminal surface lining primarily composed of proteoglycans and glycosaminoglycans, functions as a protective vascular nanobarrier [71]. Sepsis-like stimuli trigger the release of ROS, TNF-α, heparanase, and other agents, inducing glycocalyx shedding. Glycocalyx disruption can precipitate several detrimental consequences: (1) Vasomotor dysfunction, wherein endothelial structural damage impairs mechanotransduction, leading to aberrant release of NO and endothelin (ET), thereby compromising vascular regulation; (2) The proinflammatory endothelial activation, mediated by cytokines (e.g., TNF-α and IL-1α), triggers glycocalyx degradation and promotes leukocyte adhesion; (3) Metabolic dysregulation, particularly the upregulation of fatty acid metabolism; (4) Amplification of inflammation, where low-molecular-weight hyaluronan fragments elevate the expression of vascular cell adhesion molecule-1 (VCAM-1) and intercellular adhesion molecule-1 (ICAM-1), thereby polarizing macrophages and facilitating leukocyte adhesion and transcellular migration; (5) This process disrupted vascular homeostasis involving angiopoietins (Ang1/2) and their tyrosine kinase receptors (Tie1 and Tie2) axis dysregulation. ROS can cleave Tie1, thereby disrupting Tie1/Tie2 signaling. Collectively, these effects exacerbate vascular permeability, promote leakage, facilitate the efflux of intracellular substances, elevate interstitial hydrostatic pressure, worsen tissue hypoperfusion, and contribute to both inflammation and microvascular thrombosis [72].

Acute glycocalyx injury typically necessitates a recovery period of 5–7 days, with complete restoration of hemodynamic function potentially extending beyond this timeframe. The degree of glycocalyx damage can be assessed by monitoring plasma concentrations of shed glycocalyx components and endothelial cell-derived exosomal markers, such as syndecan-1 and hyaluronan [72, 73]. Additionally, real-time bedside monitoring of blood flow dynamics and microscopic evaluation of vascular thickness provide valuable insights into the integrity of the vascular barrier. Plasma levels of syndecan-1 (SDC-1) and hyaluronan exhibit a strong correlation with disease severity and DIC scores. Therapeutic interventions, including intravenous hydrocortisone administration and the infusion of fresh frozen plasma containing albumin, can attenuate glycocalyx degradation. Moreover, Tie2 receptor agonists, such as vasculotide, and VE-cadherin internalization inhibitors selectively modulate vascular permeability, thereby limiting vascular leakage [72].

### Hemostatic dysregulation

ECs’ PCD profoundly influences the hemostatic balance during sepsis. Under physiological conditions, ECs maintain the equilibrium between coagulation and fibrinolysis by expressing or interacting with coagulation-related factors. Mechanistically, ECs express tissue factor (TF), which forms a complex with factor VIIa, thereby activating factors IX and X, and initiating the coagulation cascade. Additionally, ECs synthesize tissue factor pathway inhibitor (TFPI), which binds to factor Xa, promotes fibrin deposition, and inhibits the TF-VIIa complex, thereby modulating the coagulation process [16]. Furthermore, ECs activate protein C through thrombomodulin and the endothelial protein C receptor (EPCR), which subsequently inhibits coagulation factors V (proaccelerin) and VIII (antihemophilic factor), while suppressing plasminogen activator inhibitor-1 (PAI-1), thus critically regulating fibrinolysis [74–76].

During sepsis, endotoxins and inflammatory stimuli increase the activation of a disintegrin and metalloproteinase with thrombospondin type 1 motif, member 13 (ADAMTS-13) fragments, which contribute to hypercoagulability and impaired fibrinolysis. Inflammatory mediators and substances released by activated neutrophils, such as plasmin and thrombin, can inactivate or inhibit ADAMTS-13. Concurrently, the inactivation of TFPI and protein C is diminished, while PAI-1 levels are elevated, inhibiting tissue plasminogen activator (tPA) and suppressing fibrinolysis. These cumulative effects exacerbate the development of DIC [74, 75].

Elevated plasma PAI-1 levels correlate strongly with increased mortality risk and progression to multiorgan failure. Targeted anticoagulant therapies may reduce DIC and organ failure, whereas supplementation with tPA and antithrombin III may alleviate the severity of organ dysfunction [16]. However, monotherapeutic approaches targeting specific molecular pathways have yet to demonstrate definitive success in the treatment of sepsis. Blood purification and therapeutic plasma exchange have shown some efficacy in mitigating these conditions [75]. Notably, Zhou et al. have elucidated that NINJ1 regulates platelet activation and PANoptosis in DIC during sepsis, while myricetin has been proven to reduce PANoptosis in sepsis [77].

### Inflammation and organ dysfunction

ECs’ PCD significantly contributes to local leukocyte adhesion, migration, and vascular leakage, thereby facilitating the release of inflammatory mediators and triggering localized inflammatory responses. In severe cases, this process can escalate to systemic cytokine storms and the onset of SIRS. Under normal physiological conditions, factors involved in leukocyte adhesion are embedded within the glycocalyx on the EC surface, which also forms a negatively charged layer that repels albumin adhesion. Through chemokine and adhesion molecule expression, ECs control leukocyte recruitment and transmigration. Selectins (E-, L-, and P-selectins) facilitate leukocyte adhesion and rolling, while integrins (ICAM-1 and VCAM-1) mediate firm adhesion and upregulate the transendothelial migration of leukocytes into tissues. ICAM-1, in particular, is a crucial protein in leukocyte adhesion and migration, predominantly occurring within the microcirculatory venous network. During sepsis, EC death and glycocalyx degradation expose adhesion sites for leukocytes, thereby initiating their adhesion. Concurrently, increased vascular permeability enables the migration of leukocytes into tissues, thereby propagating and disseminating inflammation. Moreover, PAMPs activate pattern recognition receptors (PRRs) and downstream inflammatory signaling pathways, inducing the synthesis of proinflammatory mediators, notably NF-κB and IL-6. These mediators, in conjunction with ROS, induce EC reprogramming, downregulate the expression of VE-cadherin and zonula occludens-1 (ZO-1), disrupt the endothelial barrier, and enhance myeloid cell trafficking and recruitment, further amplifying the inflammatory response [72].

Inflammation plays a crucial role in amplifying innate immunity, promoting tissue repair, and restoring homeostasis. However, excessive inflammatory responses can be detrimental to normal cells and tissues. During sepsis, PAMPs/DAMPs detect pathogens or tissue damage, activating immune responses and acute-phase proteins. Key cytokines (e.g., IL-1, IL-6, TNF) stimulate secondary mediators (e.g., chemokines, prostaglandins, NO), amplifying leukocyte recruitment and innate immunity while regulating vascular tone [78]. Acute-phase proteins such as C-reactive protein (CRP) serve as key biomarkers of systemic inflammation and tissue damage. Elevated circulating levels of shed endothelial adhesion molecules (E-selectin, VCAM-1, and ICAM-1) show a significant positive correlation with organ failure severity in septic patients [79]. In endotoxemia models, monoclonal antibodies targeting VCAM-1, such as synthetic amphiphile interaction-optimized Liposomes (SAINT-O-Somes), have successfully targeted ECs; however, their in vivo efficacy requires additional investigation [16].

**Fig. 4:**
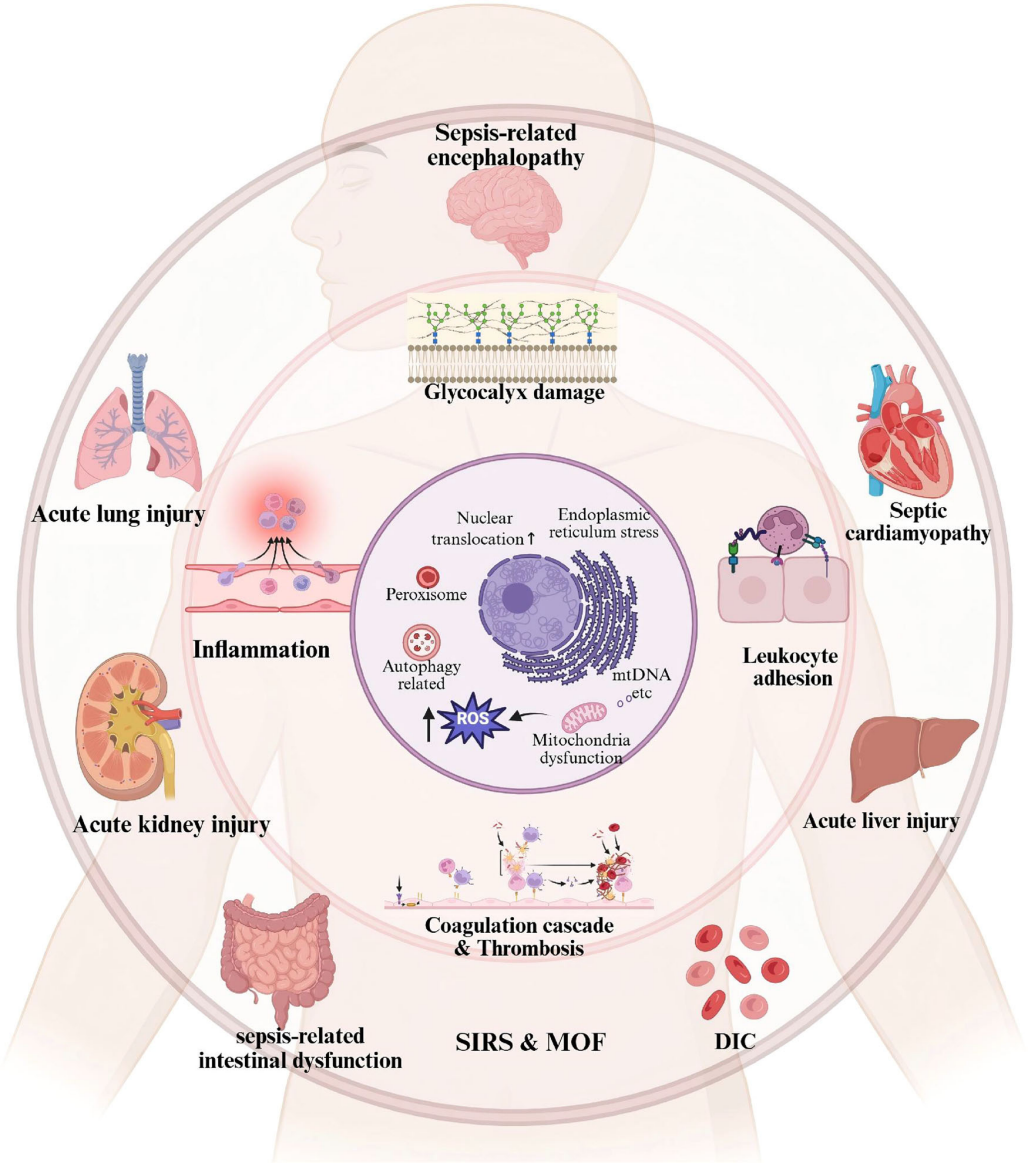
The pathophysiologic process of ECs PCD in sepsis. During sepsis, PAMPs and DAMPs activate programmed cell death pathways in endothelial cells, triggering endoplasmic reticulum stress, increased nuclear translocation and translation, peroxisome accumulation, enhanced autophagic lysosomal activity, and mitochondrial dysfunction with subsequent release of reactive oxygen species (ROS) and mitochondrial DNA (mtDNA). These components further act as DAMPs, amplifying PCD-related signaling cascades. Endothelial cell death leads to structural and functional vascular impairment, characterized by: a. degradation of the endothelial glycocalyx and reduced glycocalyx thickness; b. enhanced leukocyte adhesion and transmigration; c. coagulopathy and thrombus formation; d. focal inflammation and its systemic dissemination. At the tissue level, these alterations manifest as hemodynamic instability and impaired microcirculatory perfusion. Multi-organ involvement results in acute clinical manifestations of injury, potentially progressing to disseminated intravascular coagulation (DIC), systemic inflammatory response syndrome (SIRS), and multiple organ dysfunction syndrome (MODS). In critical cases, these pathological changes culminate in fatal outcomes.

ECs are the first responders to sepsis and can exert systemic effects on tissues and organs. Programmed endothelial cell death and vascular dysfunction play significant roles in organs such as the lungs, liver, and kidneys. However, due to anatomical and hemodynamic differences among organs, EC injury may exhibit organ-specific heterogeneity. For instance, in the kidneys, ECs produce NO and endothelin during sepsis, which influences renal perfusion and leads to tissue edema, contributing to kidney injury [80]. In the pulmonary vasculature, ECs upregulate the expression of adhesion molecules, and inflammatory factors diffuse through alveolar spaces, resulting in pulmonary edema [81]. In the liver, ECs are essential components of the sinusoidal microcirculation. In addition to mediating coagulation and leukocyte adhesion, liver ECs play key roles in antigen presentation and immune tolerance. They exhibit the highest pro-apoptotic activity in the periportal regions [7], thereby exacerbating sinusoidal edema and hypoxia [75]. Therefore, investigating the mechanisms and roles of EC injury across different organs is critical for the targeted prediction and prevention of organ dysfunction (Fig. 4).

## Potential applications of regulating ECs PCD in the diagnosis and treatment of sepsis

### ECs PCD as a novel biomarker for early hemodynamic monitoring in sepsis

Infection serves as a critical initiating factor for sepsis, while the inflammatory response represents the core pathophysiological process leading to immune dysregulation and organ injury. Etiological detection and acute-phase proteins serve as important biomarkers reflecting infectious evidence and systemic inflammatory status, respectively, guiding subsequent anti-infective and anti-inflammatory therapies, while providing a basis for severity assessment and prognosis evaluation [78, 82]. Current diagnostics like matrix-assisted laser desorption/ionization time-of-flight (MALDI-TOF) mass spectrometry and PCR/electrospray ionization (PCR/ESI)-based IRIDICA system enhance microbial analysis speed and coverage [82]. Major acute-phase proteins include: (1) humoral innate immunity markers (CRP, serum amyloid P/A, PTX3 family, complement system, mannose-binding lectin, IL-1Ra, etc.); (2) coagulation and tissue repair mediators (fibrinogen, prothrombin, urokinase-type plasminogen activator, etc.); (3) iron metabolism regulators (transferrin, ferritin, heme, hepcidin, etc.); and (4) other carrier proteins (albumin, ceruloplasmin, etc.) [78]. These proteins promote inflammation, upregulate cellular adhesion molecule expression, and mediate endothelial dysfunction [69], with well-established clinical applications. However, target organ dysfunction and failure remain challenging in clinical prediction and assessment. Moreover, inflammatory biomarkers may be influenced by transient factors like stress [83]. Therefore, identifying early biomarkers represents a crucial strategy for prognosis improvement. The latest sepsis expert consensus proposes that ideal sepsis biomarkers should possess following characteristics to evaluate disease severity, progression and guide treatment: (1) reflecting core pathophysiology with clinical utility; (2) indicating transcriptomic gene expression differences; (3) demonstrating immunometabolic alterations; (4) serving as personalized intervention and outcome predictors; and (5) providing real-time monitoring capability [2, 82]. Endothelial function monitoring enables early detection of hemodynamic changes and organ perfusion status. Programmed endothelial cell death-related molecules may serve as promising candidate biomarkers for early vascular barrier dysfunction assessment, potentially facilitating sepsis pathophysiology monitoring and personalized intervention guidance.

Notably, EC degradation represents a critical manifestation of endothelial dysfunction. Circulating markers, including heparan sulfate, sialic acid, hyaluronic acid, and syndecan-1, reliably reflect the extent of glycocalyx damage [84]. Furthermore, emerging techniques utilizing vital dyes and handheld video microscopy enable quantitative assessment of glycocalyx thickness as a surrogate for vascular permeability [71]. However, the absence of a gold standard for glycocalyx evaluation remains a significant limitation in current clinical practice. In addition to acute-phase proteins and inflammatory cytokines, Supino et al. demonstrated that the interleukin-1 receptor type 2 (IL-1R2) decoy receptor (a member of IL-1/IL-1 receptor family inhibitors) is selectively upregulated on leukocyte plasma membranes in sepsis patients and downregulated in septic shock. This receptor serves as a biomarker for macrophage differentiation and dysfunction, acute myelopoiesis, and myeloid cell activation [85]. Proteolytic enzymes, adhesion molecules (VCAM-1, ICAM-1, selectins), and coagulation factors (TF, PAI-1) serve as direct indicators of ECs’ PCD activation during sepsis. Conversely, antithrombin, prealbumin, rheumatoid factor, and the Tie2 receptor system reflect vascular barrier integrity, sensing capability, vasomotor function, and coagulation homeostasis, demonstrating significant diagnostic efficiency for sepsis mortality prediction [2]. Integrins (e.g., αvβ3, β1, β5) exhibit dual regulatory roles: (1) Promoting endothelial survival via focal adhesion kinase (FAK) and PI3K/Akt pathway-mediated caspase cascade inhibition; (2) Inducing anoikis, oxidative stress-mediated endothelial dysfunction, and pyroptosis. Soluble integrin levels quantitatively correlate with endothelial injury severity [86, 87]. However, circulating adhesion molecules can be derived from multiple cell types, including endothelial cells, leukocytes, and platelets, consequently exhibiting relatively low specificity as biomarkers of endothelial injury. Optimizing the specificity of detection methodologies represents a critical direction for future research.

Furthermore, certain endothelial injury markers have been validated for their association with disease prognosis and mortality risk. Microparticles (MPs), which are small vesicles shed from the plasma membrane, demonstrate significantly elevated circulating endothelial MP concentrations in patients with acute myocardial infarction. Additionally, CRP, asymmetric dimethylarginine (ADMA), and symmetric dimethylarginine (SDMA) contribute to endothelial vasomotor dysfunction through direct or indirect inhibition of NO signaling [84]. Elevated levels of matrix metalloproteinase-8 (MMP-8) and inter-α-inhibitor protein (IαIp) demonstrate a significant association with increased risk of organ failure [84]. Soluble thrombomodulin serves as a sensitive indicator of severe endothelial injury, while surfactant protein D correlates with alveolar-capillary membrane damage in acute lung injury. Plasma gelsolin levels reflect the host’s capacity to effectively clear released actin, representing a potential real-time biomarker for disease monitoring [2]. Furthermore, immune checkpoint markers have been validated in both clinical and experimental settings. PD-L1-expressing plasma cells exhibit potent immunosuppressive effects through inhibition of T-cell responses in septic patients and animal models, identifying this pathway as a promising therapeutic target for modulating immune cell dysfunction in sepsis [88].

Compared to other biomarkers, gene expression profiles exhibit greater stability and long-term consistency, making the monitoring of PCD-related molecules and gene expression a promising direction for sepsis diagnosis. Wang et al. employed machine learning algorithms to validate the association between apoptosis-related genes and sepsis diagnosis [89]. Yang et al. utilized gene learning and single-cell RNA-seq analysis to identify propionate-related metabolic genes as potential biomarkers for sepsis progression and therapeutic targets, providing insights into the mechanisms underlying metabolic dysregulation in sepsis [83]. Although these findings still require further in vitro/in vivo experimental validation and clinical confirmation, they offer valuable directions for future research.

Regarding microcirculatory monitoring, current guidelines emphasize the importance of real-time hemodynamic assessment, as sepsis induces alterations in microcirculatory flow patterns, reduced vascular density, and a decreased proportion of perfused vessels. These microvascular disturbances are closely associated with impaired tissue perfusion, the severity of organ failure, and increased mortality risk. Table 2 provides a summary of the currently available clinical tools for evaluating microcirculation. The critical role of ECs’ PCD in the pathogenesis of sepsis suggests its diagnostic significance. The molecular signature associated with PCD, alongside genetic markers, has been shown to correlate with sepsis diagnosis through the application of machine learning algorithms [89].

**Table 2:** Monitoring modalities and parameters for microcirculatory status assessment.

Monitoring modality	Measured parameters
Phenotypic Observation	Cutaneous mottling/marbling; Prolonged capillary refill time; Peripheral gangrene
Circulatory Symptoms	Oliguria
Orthogonal Polarization Spectral Imaging	Sublingual microcirculation visualization
Near-Infrared Spectroscopy (NIRS)	Non-invasive hemoglobin oxygen saturation monitoring
Iontophoretic Acetylcholine Delivery with Laser Doppler	Blood flow index and endothelium-dependent vasodilatory reserve recording
Blood Cell Morphology	Reduced RBC deformability (associated with poor prognosis)

### ECs' PCD provides novel therapeutic intervention targets for sepsis

The current standardized treatment for sepsis primarily involves vasoactive agent administration (e.g., norepinephrine for hemodynamic stabilization), inotropic support, anticoagulation therapy, fluid resuscitation, and pathogen-directed antimicrobial treatment [90]. While these measures provide essential supportive care, they remain largely symptomatic and have yet to address the fundamental pathophysiological mechanisms. Recent research has identified EC’s PCD signaling pathways and their critical molecular mediators as promising therapeutic targets in sepsis pathogenesis. Notably, several targeted agents and small-molecule inhibitors directed against these pathways have emerged as potential disease-modifying interventions. Multiple small-molecule compounds and active components derived from traditional Chinese medicine have demonstrated significant pharmacodynamic effects in preclinical sepsis models [37].

While drugs targeting core PCD molecules have demonstrated experimental and/or clinical validation across various disease models, their therapeutic efficacy in sepsis requires further investigation. In the context of apoptosis inhibition, the pan-caspase inhibitor emricasan has exhibited anti-apoptotic effects in cochlear and hepatocyte models; however, its impact on septic ECs remains unexamined [91]. Interleukin-35 has shown anti-apoptotic activity through the activation of STAT1/4 signaling. Regarding necroptosis modulation, the RIPK1-specific inhibitor Necrostatin-1 mitigates LPS-induced liver injury in murine models. Additionally, metformin (via AMPK/STAT3 pathway), rapamycin, and quercetin display anti-necroptotic and organ-protective effects [92]. For pyroptosis regulation, Erlotinib inhibits TLR4-mediated parthanatos [33], and rapamycin suppresses pyroptosis via mTOR inhibition, potentially modulating its effects. In terms of ferroptosis, Dexmedetomidine, irisin, and ferrostatin-1 have shown protective effects in preclinical studies, while dexrazoxane provides cardioprotection through HMGB1 modulation, though its application in sepsis remains to be validated [51]. Mitochondrial dysfunction serves as a critical mediator of endothelial cell death in sepsis, making mitochondrial homeostasis maintenance a promising therapeutic strategy. Mitochondria-targeted antioxidants (e.g., MitoQ, MitoVitE, MitoTempol) demonstrate superior anti-inflammatory effects compared to non-targeted antioxidants. Urolithin A (UA) promotes mitophagy, while compounds like Mdivi-1, P110, and irisin inhibit mitochondrial fission, collectively exerting cytoprotective effects and improving outcomes. Cyclosporine A (CsA), clinically used as an immunosuppressant, exhibits sepsis-modulating properties through: Cyp-D interaction reducing DAMPs release, calcineurin inhibition preventing nuclear factors of activated T cells (NFAT) and NF-κB nuclear translocation, downregulation of iNOS, proinflammatory cytokines, and prostaglandins. Metformin displays dose-dependent mechanisms: low-dose metformin activates AMPK via the lysosomal pathway, inhibiting mTORC1 and promoting mitophagy to suppress NLRP3 inflammasome activation. High-dose metformin inhibits complex I of the respiratory chain, reducing ATP production and oxidative mtDNA generation, thereby attenuating pyroptosis and inflammation [18]. The endoplasmic reticulum sigma-1 receptor (S1R) serves as a critical regulator of ER stress-mediated inflammation. Fluvoxamine, an S1R-selective serotonin reuptake inhibitor with antidepressant and anti-inflammatory properties, demonstrates protective effects against lethal septic shock in murine models. Mesenchymal stem cell (MSC) transplantation exhibits multimodal therapeutic potential by reducing systemic cytokine levels, restoring mitochondrial and metabolic function in satellite cells, and preventing sepsis-associated neuromuscular complications [93]. Synthetic HNPs function as histone-chelating agents through selective neutralization of circulating histones (key DAMPs causing endothelial dysfunction), peripheral sequestration of histones to inhibit platelet aggregation, and attenuation of pulmonary leukocyte migration, collectively improving outcomes in experimental sepsis models [94].

In contrast to drugs targeting a single molecular entity within ECs PCD signaling pathways, natural food-derived plant extracts and traditional Chinese medicine (TCM) herbs exert broader pharmacological effects and exhibit more comprehensive regulatory actions on the body. Epigallocatechin-3-gallate (EGCG), a major polyphenol in green tea, and L-theanine, an amino acid (γ-glutamyl ethylamide) and glutamate derivative, exhibit vasodilatory regulatory functions. EGCG activates the Fyn/PI3K/Akt/eNOS (endothelial nitric oxide synthase) pathway in endothelial cells to upregulate NO levels, while also increasing heme oxygenase-1 (HO-1) expression to achieve antihypertensive effects. L-theanine acts as a mood stabilizer and neuroprotective agent, similarly activating eNOS to enhance NO production and promote vasodilation. Mechanistically, EGCG inhibits TNF-α-induced PAI-1 through ERK1/2 phosphorylation in endothelial cells, attenuates TNF-α-stimulated monocyte chemoattractant protein 1 (MCP1) production, reduces Akt phosphorylation, and suppresses MAPK/NF-κB activation. In sepsis models, both EGCG and L-theanine downregulate endothelial expression of adhesion molecules (VCAM-1, microtubule-associated protein 1 light chain 3/3B(LC3/LC3B)) while inhibiting TNF-α-induced transcription of apoptotic genes (e.g., caspase-9) and caspase activity, thereby exerting anti-apoptotic effects [95]. Liangge San (LGS), a traditional Chinese herbal formulation for treating acute pathogen-induced syndromes of the upper and middle jiao, contains bioactive components including luteolin and wogonin that may ameliorate LPS-induced acute lung injury through multiple mechanisms. These compounds potentially upregulate miR-21 expression while simultaneously inhibiting both the STAT3 signaling pathway and the p38-MAPK-NF-κB cascade. The formulation demonstrates significant immunomodulatory effects by suppressing macrophage production of proinflammatory cytokines (IL-6, TNF-α, and IL-1β), thereby reducing oxidative stress and inflammatory responses [96, 97]. LGS modulates key metabolic pathways (glycine/serine/threonine, cysteine/methionine, inositol phosphate, and TCA cycle metabolites), conferring anti-apoptotic effects [98]. Qishenyiqi Dripping Pills (QSYC), a clinically recommended treatment for coronary heart disease with demonstrated effects in replenishing qi, promoting blood circulation, and relieving pain, has been shown to maintain pulmonary vascular barrier integrity and ameliorate lung injury in sepsis through mechanisms involving suppression of the RAGE pathway and targeted inhibition of cyclooxygenase-2 (COX-2) in endothelial cells, thereby preventing ferroptosis in CLP-induced septic models [36]. The active components in Babao Dan (BBD), a traditional medicine used for treating viral hepatitis and cholecystitis, including bile acids and saponins, exert anti-pyroptotic effects by suppressing NLRP3-regulated inflammasome activation [99]. Additionally, the anti-cell death effects of numerous active components and monomeric extracts derived from traditional Chinese medicines have been experimentally validated: astragaloside IV (AS-IV) counteracts ferroptosis in ECs by reversing the LPC-induced elevation of iron and lipid peroxides, as well as mitochondrial shrinkage [37]. Artesunate (AS) enhances the expression of anti-ferroptotic systems, such as nuclear factor erythroid 2-related factor 2 (Nrf2) and HO-1, while also mitigating neutrophil infiltration and pathological damage in lung tissue [51].

Nevertheless, broad-spectrum PCD inhibitors and natural extracts encounter several challenges, including imprecise targeting, unpredictable efficacy, and limited bioavailability/biostability. To overcome these obstacles, advanced drug delivery systems, such as nanoencapsulation and liposomal formulations, offer promising solutions. For example, Yan et al. developed an engineered capsule containing the PARP1 inhibitor olaparib (OLA), designed for targeted intestinal release [40]. Nicotinamide adenine dinucleotide (NAD), while functioning as an immunomodulatory agent, exhibits limited bioavailability; however, its encapsulation within nanoparticle delivery systems enables direct transport and replenishment of NAD(H), thereby enhancing cellular energy metabolism, suppressing both apoptosis and pyroptosis, and consequently maintaining immune homeostasis and vascular function, ultimately leading to improved outcomes in experimental sepsis models [100]. Alternatively, non-coding RNAs-including long non-coding RNAs (lncRNAs), circular RNAs (circRNAs), and microRNAs (miRNAs) - precisely regulate cellular mechanisms. Acting as competitive endogenous RNAs (ceRNAs), these molecules control endothelial cell apoptosis in sepsis, suggesting their therapeutic potential. For instance, taurine-upregulated gene 1(TUG1) mitigates inflammation and apoptosis via targeting miR-34b-5p (downstream effector: GAB1) or miR-27a-3p (downstream effector: SLIT2) during sepsis. CircRNA C3P1 demonstrates protective effects in pulmonary microvascular endothelial cells (PMVECs) by exerting both anti-inflammatory and anti-apoptotic actions through miR-21 targeting. Conversely, miR-92a promotes LPS-induced apoptosis in human PMVECs (HPMECs) via suppression of the AKT/mTOR pathway, thereby accelerating ARDS progression. MiR-15b-5p and SIRT4 together inhibit apoptosis and inflammatory responses [101]. By targeting stress-associated endoplasmic reticulum Protein 1(SERP1), miR-1-3p aggravates endothelial dysfunction via impaired proliferation, enhanced apoptosis, cytoskeletal remodeling, and elevated permeability, critically contributing to ALI-associated vascular barrier failure [102]. Furthermore, adipose-derived stem cell (ADSC)-derived exosomal miR-125b-5p protects against sepsis-induced ferroptosis in pulmonary microvascular ECs via Keap1/Nrf2/GPX4 pathway, mitigating sepsis-associated ALI [103]. Finally, decoy receptors, such as EphA4-Fc (a pan-ephrin inhibitor), help preserve endothelial barrier integrity by preventing TNF-α/ephrin-A1-induced degradation of junctional proteins, notably VE-cadherin [104]. Table 3 provides a synopsis of the pertinent advancements in drug targets for ECs PCD within the context of sepsis.

**Table 3:** Programmed endothelial cell death in sepsis: drug targets and progression.

PCD	Drugs	Targets and signaling pathways	Models	Reference
Apoptosis	Resveratrol	Upregulate SIRT1, increase antioxidant production, reduce mitochondrial-related apoptotic signaling pathways, and alleviate ROS-induced damage.	LPS-treated human bronchial epithelial cells	[9, 25, 91, 92, 95, 98, 101, 102]
	Adiponectin	Reduce GRP78, CHOP, and caspase-12; attenuate the IRE1α pathway of endoplasmic reticulum stress and ROS.	CLP-Rat；LPS-HUVEC	
	Dihydroartemisinin	Inhibit caspase-3, alleviate inflammation.	LPS-mouse	
	Ginkgolide Aglycone	Upregulate SIRT1, block inflammation.	LPS-mouse;LPS-HK-2	
	Geniposide	Activate PPARγ; increase Bcl-2, reduce Bax and caspase-3; decrease vascular permeability, inflammation, and oxidative stress.	CLP-mouse;LPS-HK-2	
	Nefilin	Upregulate Klotho, reduce inflammation.	LPS-mouse;LPS-NRK52E	
	Dexmedetomidine	Reduce MALAT1, ALKBH5, and inflammation.	LPS-HK-2	
	Arbutin	Regulate the PI3K/Akt/Nrf2 pathway; Increase Bcl-2, reduce Bax, caspase-3, and caspase-9.	LPS-Rat;LPS-NRK-52e	
	DL-Propargylglycine	Reduce Bax, P53, and inflammation.	LPS-RAW	
	Interleukin-35,	Activate STAT1/4 to exert anti-apoptotic effects.	LPS-mouse	
	SW033291	Inhibit 15-PGDH, downregulate lipid peroxidation; increase Bcl-2, downregulate Fas, caspase-3, and caspase-8.	LPS-mouse	
	SPA0355	Inhibit the P53 signaling pathway and inflammation.	LPS-mouse	
	TMP195 series	Inhibit class IIa HDACs, increase Bax and cleaved caspase-3, reduce Bcl-2 and bone morphogenetic protein-7.	LPS-mouse; LPS- RTEC	
	AICAR or Metformin	Increase Sirt3, activate AMPK, and restore mitochondrial function.	CLP-mouse;LPS + HMGB1-HK-2	
	Paricalcitol	Increase the vitamin D receptor and Bcl-2, and reduce cleaved caspase-3.	LPS-mouse	
	Epigallocatechin-3-gallate	Inhibit Sema3A; increase Bcl-2; reduce Bax and cleaved caspase-3.	LPS-mouse;LPS-NRK52E	
	Liang-Ge-Tang	Suppress inflammation, oxidative stress, apoptosis, and regulate amino acid metabolism.	CLP-Rat	
	Non-coding RNA	lncRNA taurine-upregulated TUG1-miR-34b-5p and GAB1/miR-27a-3p and SLIT2; LUADT1-miR-195-upregulate Pim-1-inhibit apoptosis; CirC3P1-miR-21 anti-inflammatory and anti-apoptotic; miR-92a-AKT/mTOR; miR-1-3p-SERP1 promotes ALI development; miR-15b-5p-SIRT4 inhibits apoptosis and inflammatory responses.	LPS-mouse；LPS-HPCAEC；LPS-HPMEC	
Necroptosis	Bosentan	Block ET receptor; reduce TLR4, MyD88, and RIPK3 phosphorylation; improve cell migration properties.	LPS-BMVEC	[23, 37, 42, 92]
	Necrostatin-1	Inhibit RIPK1; reduce LC3-II and p62.	LPS-mouse	
	Cyclosporin A, Rapamycin, Heat Shock Protein 90 Inhibitors, etc. Patchoulol	-	LPS-mouse	
	Patchoulol	-	-	
	Stress-induced protein Sestrin2	Inhibit MLKL.	LPS-DC2.4	
	Metformin	Inhibit renal injury in lupus nephritis through suppression of the AMPK/STAT3 pathway	in lupus nephritis	
Pyroptosis	Isocyanic acid	Reduce NLRP3, caspase-1, IL-1β, IL-18, TLR4, and MyD88; inhibit phosphorylation of AKT and PKC-α/δ.	LPS-RAW264.7; LPS-mouse	[18, 23, 40, 64]
	CC-5013 series	Inhibit the TNF-α/HMGB1 signaling pathway and inflammation.	LPS/D-Gal, mouse;LPS-M1 Macrophages	
	Samotolisib	Inhibit the PI3K/AKT/mTOR pathway, Nedd4, and caspase-11.	LPS-mouse;LPS-RAW264.7	
	Mdivi-1	Inhibit DLP1; reduce NLRP3, cleaved caspase-1, GSDMD, IL-1β, and IL-18.	LPS-mouse; LPS-mouse RTEC	
	Znpp	Inhibit HO-1/PINK1, inflammation, and oxidative stress; regulate mitochondrial fusion/fission.	LPS-Rat	
	AC-YVAD-CMK	Inhibit caspase-1; reduce NLRP-1, IL-1β, IL-18, and GSDMD.	CLP-mouse	
	HU308	Increase cannabinoid receptor 2; reduce NLRP3, caspase-1, and GSDMD activation.	CLP-mouse;LPS-BMDM	
	C16(C13H8N4OS)	Inhibit protein kinase R, reduce ASC, NLRP3, and caspase-1.	LPS-mouse	
	Thymoquinone	Reduce NLRP3, caspase-1, caspase-3, caspase-8, and inflammation.	CLP-mouse	
	N-Acetyl-L-cysteine	Scavenge ROS, reduce cleaved caspase-1, NLRP3, and cleaved GSDMD.	LPS-HUVEC	
	Metformin	High-dose: inhibits complex I of the respiratory chain, reducing ATP and mtDNA, reduce NLRP3.	-	
Parthanatos	Erlotinib	Inhibit TLR4	-	[39]
Ferroptosis	Astragaloside IV (AAS-IV)	Reverse iron and lipid peroxide elevation, reverse mitochondrial atrophy	-	[37, 103]
	miR-125b-5p	Regulate Keap1/Nrf2/GPX4 to alleviate pulmonary microvascular endothelial cell death	LPS-PMVEC	
PANoptosis	Ursodeoxycholic acid	Block PANoptosis via the STING pathway, alleviate sepsis-induced lung injury	LPS-mice	[61, 69]
	Xiao Chai Hu Tang (Minor Bupleurum Decoction)	Inhibit ZBP1 to block PANoptosis, reduce LPS-induced cardiomyocyte injury	LPS-mice; LPS-H9c2 cells	

## Conclusion and prospects

This review comprehensively analyzes recent breakthroughs in ECs’ PCD mechanisms and their contributions to sepsis pathogenesis. We systematically delineate the pathophysiological continuum from initial ECs’ death events to the development of profound endothelial dysfunction, a critical determinant of sepsis progression. Our analysis highlights the emerging diagnostic and therapeutic potential of targeting ECs’ PCD pathways in sepsis management.

Nevertheless, several pivotal challenges persist in this field. Firstly, current investigations remain largely confined to isolated PCD pathways, neglecting the complex interplay and phenotypic plasticity between distinct death modalities. The newly proposed PANoptosis paradigm, while conceptually promising, requires rigorous mechanistic validation in ECs, particularly regarding the formation and regulation of PANoptotic complexes during sepsis. Secondly, although ECs’ PCD is known to drive microvascular dysfunction through multiple mechanisms, including increased vascular permeability, barrier disruption, coagulopathy, and inflammatory amplification, the precise molecular cascades and signaling networks orchestrating these effects remain incompletely characterized. Finally, while numerous ECs’ PCD-related biomarkers and molecular targets have been identified, their clinical translation necessitates robust validation through multicenter randomized controlled trials to establish diagnostic reliability and therapeutic efficacy.

Looking forward, the convergence of single-cell technologies, advanced molecular imaging, and systems biology approaches offers unprecedented opportunities to decipher the spatiotemporal regulation of ECs’ death programs, develop precision therapeutics targeting PANoptotic signaling nodes, and establish biomarker-guided treatment algorithms. These innovations hold significant potential to transform sepsis management by preserving endothelial integrity and improving patient outcomes.
